# Immunological Molecular Responses of Human Retinal Pigment Epithelial Cells to Infection With *Toxoplasma gondii*

**DOI:** 10.3389/fimmu.2019.00708

**Published:** 2019-05-01

**Authors:** Shervi Lie, Elise Rochet, Erik Segerdell, Yuefang Ma, Liam M. Ashander, Audra M. A. Shadforth, Timothy A. Blenkinsop, Michael Z. Michael, Binoy Appukuttan, Beth Wilmot, Justine R. Smith

**Affiliations:** ^1^Eye and Vision Health, Flinders University College of Medicine and Public Health, Adelaide, SA, Australia; ^2^Department of Biostatistics, Oregon Health and Sciences University, Portland, OR, United States; ^3^Queensland Eye Institute, Brisbane, QLD, Australia; ^4^School of Biomedical Science, Queensland University of Technology, Brisbane, QLD, Australia; ^5^Departments of Cell, Developmental and Regenerative Biology, and Ophthalmology, Icahn School of Medicine at Mount Sinai, New York, NY, United States; ^6^Flinders Centre for Innovation in Cancer, Flinders University College of Medicine and Public Health, Adelaide, SA, Australia

**Keywords:** human, retinal pigment epithelium, infection, *Toxoplasma gondii*, RNA-sequencing, gene ontology, pathway, network

## Abstract

Ocular toxoplasmosis is the commonest clinical manifestation of infection with obligate intracellular parasite, *Toxoplasma gondii*. Active ocular toxoplasmosis is characterized by replication of *T. gondii* tachyzoites in the retina, with reactive inflammation. The multifunctional retinal pigment epithelium is a key target cell population for *T. gondii*. Since the global gene expression profile is germane to understanding molecular involvements of retinal pigment epithelial cells in ocular toxoplasmosis, we performed RNA-Sequencing (RNA-Seq) of human cells following infection with *T. gondii* tachyzoites. Primary cell isolates from eyes of cadaveric donors (*n* = 3), and the ARPE-19 human retinal pigment epithelial cell line, were infected for 24 h with GT-1 strain *T. gondii* tachyzoites (multiplicity of infection = 5) or incubated uninfected as control. Total and small RNA were extracted from cells and sequenced on the Illumina NextSeq 500 platform; results were aligned to the human hg19 reference sequence. Multidimensional scaling showed good separation between transcriptomes of infected and uninfected primary cell isolates, which were compared in edgeR software. This differential expression analysis revealed a sizeable response in the total RNA transcriptome—with significantly differentially expressed genes totaling 7,234 (28.9% of assigned transcripts)—but very limited changes in the small RNA transcriptome—totaling 30 (0.35% of assigned transcripts) and including 8 microRNA. Gene ontology and pathway enrichment analyses of differentially expressed total RNA in CAMERA software, identified a strong immunologic transcriptomic signature. We conducted RT-qPCR for 26 immune response-related protein-coding and long non-coding transcripts in epithelial cell isolates from different cadaveric donors (*n* = 3), extracted by a different isolation protocol but similarly infected with *T. gondii*, to confirm immunological activity of infected cells. For microRNA, increases in miR-146b and miR-212 were detected by RT-qPCR in 2 and 3 of these independent cell isolates. Biological network analysis in the InnateDB platform, including 735 annotated differentially expressed genes plus 2,046 first-order interactors, identified 10 contextural hubs and 5 subnetworks in the transcriptomic immune response of cells to *T. gondii*. Our observations provide a solid base for future studies of molecular and cellular interactions between *T. gondii* and the human retinal pigment epithelium to illuminate mechanisms of ocular toxoplasmosis.

## Introduction

Toxoplasmosis is an infectious disease caused by the obligate intracellular Apicomplexan protozoan, *Toxoplasma gondii* (1). Approximately one-third of the global population is infected with the parasite, including persons in both industrialized and developing nations (2). In humans, *T. gondii* exhibits tropism for the central nervous system (3). The most frequent clinical manifestation of infection is an inflammatory eye disease commonly referred to as ocular toxoplasmosis (4). In Brazil, where extremely high rates of *T. gondii* infection are recorded, up to 17% of the population have ocular toxoplasmosis, while in countries with relatively low rates of infection, such as the United States, it is estimated that approximately 2% of persons have this condition (5). Toxoplasmosis also may be manifest as various neurological deficits, and associations between *T. gondii* infection and mental health—including psychiatric diseases and risk-taking behaviors—have been recognized recently (6, 7). Clinical disease is more common and more aggressive when contracted *in utero*, as well as in aged persons and individuals with compromised immunity (8–10).

Active ocular toxoplasmosis is characterized pathologically by rapid replication of the tachyzoite form of *T. gondii* within the retina, plus reactive inflammation (4, 5). Tachyzoite replication ultimately destroys the host cell, and an affected eye demonstrates necrotic retinitis, often associated with vitritis and choroiditis. Typically ocular toxoplasmosis is active for 6–8 weeks, after which time, the parasite converts to the bradyzoite form, which demonstrates limited replication and low immunogenicity, and the retinal inflammation resolves with scarring. Clinicopathological correlations show that the retinal pigment epithelium, which lies between the neural retina and the choroid, is a key target cell population for *T. gondii* (11, 12). This epithelial monolayer contributes to the blood-retinal barrier, and performs multiple diverse functions, including: light absorption, production of growth factors and signaling molecules, control of subretinal ion homeostasis, all-trans retinal re-isomerization during the visual cycle, phagocytosis of photoreceptor debris, and maintenance of immune privilege in the posterior eye (13). Over several decades, multiple research groups have described individual molecular responses of human retinal pigment epithelial cells to infection with *T. gondii* (14–18). The transcriptome of the infected retinal pigment epithelium has not been reported, however. Although ocular fluid is collected commonly in the clinic when diagnosing a retinitis (19), the vision-critical retinal pigment epithelium is not biopsied for clinical purposes, and infection of the human retinal pigment epithelial cell therefore must be studied *in vitro* with cultured cells (20).

Since the global gene expression profile is germane to understanding molecular involvements of the retinal pigment epithelium in ocular toxoplasmosis, we performed RNA-Sequencing (RNA-Seq) of primary human cell isolates following infection with *T. gondii* tachyzoites. The commercially available ARPE-19 human retinal pigment epithelial cell line (21), which is widely used to study the epithelium as it has similar morphology, biochemical, and functional properties—including barrier formation, phagocytosis, and immunological activities—(21–24) was infected and studied in parallel. Infections were performed using GT-1 strain *T. gondii*. This is a naturally occurring, virulent parasite strain, which is classified genetically as a Type I or Haplogroup 1 strain (25, 26). Infections were performed for 24 h, to coincide with established tachyzoite replication and reflect the host molecular response to this process (27, 28). *In silico* pathway and network analyses of the transcriptomes demonstrated immunological activation of *T. gondii*-infected retinal pigment epithelial cells, which was confirmed by reverse transcription-quantitative real-time polymerase chain reaction (RT-qPCR) in cell isolates from unrelated donors.

## Materials and Methods

### Study Design

Primary human retinal pigment epithelial cell isolates, generated from eyes of three cadaveric donors, and the ARPE-19 human retinal pigment epithelial cell line, were infected with GT-1 strain *T. gondii* tachyzoites (multiplicity of infection, MOI = 5) or incubated without infection. After 24 h, total and small RNA were extracted from the cultures, and subsequently sequenced using the Illumina NextSeq 500 platform up to 50 million reads per sample for both total and small RNA. Data were aligned to the human hg19 and *T. gondii* reference sequences. Genes that were differentially expressed between *T. gondii*-infected and uninfected primary human retinal pigment epithelial cell samples were identified using edgeR software. Based on the results of this analysis, and of follow-up gene ontology and pathway enrichment analyses performed with CAMERA software, we sought to validate a strong immune response transcriptomic signature of infected cells. Primary human retinal pigment epithelial cell isolates, generated from eyes of three additional cadaveric donors using a different isolation and culture protocol, were similarly infected with *T. gondii* or not infected, and processed to extract total and small RNA. Multiple immune response-associated protein-coding and long non-coding RNA were validated by RT-qPCR. Immunologically focused network analyses of differentially expressed transcripts were identified using the InnateDB platform.

### Human Subjects

Human subjects research was approved by the Southern Adelaide Clinical Human Research Ethics Committee (protocol number: 175.13). Human cadaver donor eyes were obtained from the Eye Bank of South Australia (Adelaide, Australia). Human cadaver eye donors ranged from 50 to 77 years at death, and the time from death to processing of the eyecup averaged 14.5 h.

### Cell Culture

There are multiple published methods for the isolation and culture of retinal pigment epithelial cells from human eyes (29). Different methods have the potential to impact molecular phenotype, and cells were isolated by different techniques for RNA-Seq and RT-qPCR studies, since the results obtained with the latter would be used to validate the results obtained with the former. Residual anterior segment tissues, vitreous and neural retina were carefully removed from posterior eyecups ahead of cell isolation. For each method, phenotype of the isolated cell was assessed immunocytochemically, on expression of retinal pigment epithelial markers (i.e., cytokeratin-8, retinal pigment epithelium-specific protein 65, cellular retinaldehyde-binding protein, and zonula occludens 1), but not α-smooth muscle actin, which is indicative of mesenchymal differentiation.

For the first method (used for RNA-Seq), the eyecup was filled with 0.5 mg/mL collagenase IA and 0.5 mg/mL collagenase IV (Sigma-Aldrich Merck, St Loius, MO) in Hanks balanced salt solution (HBSS) and incubated for 40 min at 37°C and 5% CO_2_ in air. Subsequently the enzymatic solution was replaced with 1:1 Dulbecco's modified Eagle's medium (DMEM):F12 medium (Thermo Fisher Scientific-Gibco, Grand Island, NY; catalog number: 10565) supplemented with 1x Insulin-Transferrin-Selenium (ITS) supplement (Thermo Fisher Scientific-Gibco), 100 U/mL Penicillin-Streptomycin (Thermo Fisher Scientific-GIBCO), 1 μg/mL amphotericin (Sigma-Aldrich Merck) and 10% heat-inactivated fetal bovine serum (FBS, Bovogen Biologicals, Keilor East, Australia, or GE Healthcare-HyClone, Logan, UT), and the retinal pigment epithelium was scraped from the interior of the eyecup. Cells in suspension were pelleted by centrifugation and resuspended in fresh medium, and the final suspension was mixed to break cell sheets. Cells were seeded on 35 mm diameter collagen IV-coated dishes (Corning, Corning, NY) and incubated at 37°C and 5% CO_2_ in air. After 1 week, concentration of the ITS supplement was halved. Following initial growth, cells were passaged by trypsinization into 6-well plates (9.6 cm^2^ diameter wells) and further expanded in DMEM:F12 medium with 0.5x ITS supplement and up to 5% FBS, using brief treatment with 0.05% trypsin to remove any contaminating cells. When confluent, cultured cells were frozen in liquid nitrogen ahead of infection (at passage 3) and study by RNA-Seq.

For the second method (used for RT-qPCR), the retinal pigment epithelium-choroid was dissected from posterior eyecups and incubated with 0.5 mg/mL collagenase IA and 0.5 mg/mL collagenase IV in HBSS for 30 min at 37°C and 5% CO_2_ in air and subsequently rinsed with Dulbecco's phosphate buffered saline (PBS) with 2% FBS. Sheets of retinal pigment epithelium were gently scrapped off Bruch's membrane with a spatula at the dissecting microscope and suspended in fresh PBS with 2% FBS. Suspended cell sheets were pelleted by centrifugation and resuspended in fresh medium, and subsequently layered onto 15% sucrose solution in DMEM:F12 medium with 10% FBS, and centrifuged to collect the cell sheets. Sheets were resuspended in 50% Minimum Essential Medium Eagle alpha modification (with sodium bicarbonate) [MEM], 25% DMEM and 25% F-12 with 1x N1 Medium Supplement, 1x Non-Essential Amino Acids Solution, 1x GlutaMAX Supplement, 0.25 mg/mL taurine, 0.02 μg/mL hydrocortisone, 0.013 ng/mL 3,3',5-triiodo-L-thyronine sodium, 100 U/mL Penicillin-Streptomycin (all obtained from Sigma-Aldrich or Thermo Fisher Scientific-GIBCO), and 10% FBS. Sheets were distributed in 6 cm diameter tissue culture dishes, and cultured at 37°C and 5% CO_2_ in air for 24 h, after which the FBS supplement was reduced to 2%. Contaminating cells were removed from cultures by scrapping and washing with PBS. Cells were moved to 12-well plates by trypsinzation for infection and were rested at confluent for 2 weeks ahead of infection (at passage 1) and study by RT-qPCR.

The ARPE-19 human retinal pigment epithelial cell line (American Type Culture Collection, Manassas, VA; catalog number CRL-2302) (21) was cultured in DMEM:F12 medium supplemented with heat-inactivated 10% FBS (reduced to 5% for parasite infections) at 37°C and 5% CO_2_ in air.

### Parasite Culture

The GT-1 common, natural *T. gondii* strain (gift of Dr. Jitender P. Dubey, U.S. Department of Agriculture, Beltsville, MD; and Dr. L. David Sibley, Washington University, St Louis, MO) (25) was maintained in tachyzoite form by serial passage in confluent human neonatal dermal fibroblast monolayers (Cascade Biologics, Portland, OR) in Dulbecco's modified Eagle's medium (Thermo Fisher Scientific-Gibco, Grand Island, NY; catalog number: 12100), supplemented with 44 mM sodium bicarbonate and 1% heat-inactivated FBS, at 37°C and 5% CO_2_ in air. Parasite viability was evaluated by plaque assay in fibroblast monolayers and required to be at least 15%, which is the expected viability of freshly egressed GT-1 strain tachyzoites (30). This *in vitro* research with *T. gondii* was approved by the Flinders University Institutional Biosafety Committee (Microbiological Dealing approval number 2013-08).

### Infection of Retinal Pigment Epithelial Cells With *T. gondii*

Confluent monolayers of retinal pigment epithelial cells were infected with *T. gondii* tachyzoites at MOI of 5 in 6-well plates for RNA-Seq or in 12-well plates (3.8 cm^2^ diameter wells) for RT-qPCR. Cells and tachyzoites were incubated in culture-specific medium, including the heat-inactivated FBS, at 37°C and 5% CO_2_ in air. After 4 h, the monolayers were washed 4-times with medium to remove extracellular tachyzoites, and the infected cultures were returned to incubation in fresh medium to a total of 24 h.

### RNA Isolation

Total RNA was extracted from retinal pigment epithelial cells with TRIzol Reagent (Thermo Fisher Scientific-Ambion, Carlsbad, CA), used according to the retailer's instructions, and frozen at −80°C. RNA concentration was determined by spectrophotometry on the Qubit 2.0 Fluorometer (Thermo Fisher Scientific-Invitrogen) for RNA-Seq and on the NanoDrop 2000 (Thermo Fisher Scientific, Wilmington, DE) for RT-qPCR. Integrity of the RNA for RNA-Seq was verified on the 2100 Bioanalyzer (Agilent Technologies, Waldbronn, Germany).

### RNA-Sequencing

Total and small RNA were converted to cDNA libraries, using the TruSeq Stranded Total RNA Kit with Ribo-Zero Gold and the TruSeq Small RNA Library Prep Kit, respectively (both from Illumina, San Diego, CA), in accordance with the manufacturer's instructions with one exception: for small RNA, cDNA purification was performed using Pippin Prep (Sage Science, Beverly, MA), as described by the manufacturer. RNA samples were diluted to 1 pM ahead of sequencing on the Illumina NextSeq 500, which was performed for total RNA with the NextSeq 500/550 High Output Kit (Illumina, 150 cycles) run as paired-end 2 × 75 bp, and for small RNA with the NextSeq 500/550 High Output Kit (Illumina, 75 cycles) run as single-read 1 × 75 bp. The PhiX Control v3 library (Illumina) was used as a sequencing control.

### Total RNA Reverse Transcription and Quantitative Real-Time Polymerase Chain Reaction

Reverse transcription for total RNA was performed using the iScript Reverse Transcription Supermix for RT-qPCR (Bio-Rad, Hercules, CA), with 500 ng of RNA template yielding 20 μL cDNA. Quantitative PCR was performed on the CFX Connect Real-Time PCR Detection System (Bio-Rad) using 2 μL of cDNA diluted to 1:10, 4 μL of iQ SYBRGreen Supermix (Bio-Rad), 1.5 μL each of 20 μM forward and reverse primers, and 11 μL of nuclease-free water for each reaction. Primer sequences and product sizes for all transcripts are presented in Supplementary Table 1. Amplification consisted of: a pre-cycling hold at 95°C for 5 min; 40 cycles of denaturation for 30 s at 95°C; annealing for 30 s at 60°C; extension for 30 s at 72°C; and a post-extension hold at 75°C for 1 s. A melting curve, representing a 1-s hold at every 0.5°C between 70 and 95°C, was generated to confirm that a single peak was produced for each primer set. Standard curves, produced with serially diluted product, confirmed PCR efficiency of 80% or greater. Size of PCR product was confirmed by electrophoresis on 2% agarose gel. The cycle threshold was measured, with Cq determination mode set to regression. Relative expression was determined using the Pfaffl mathematical model, and normalized to two stable reference genes—ribosomal protein lateral stalk subunit P0 (RPLP0) and peptidylprolyl isomerase A (PPIA). Relative expression of transcript in *T. gondii*-infected and uninfected retinal pigment epithelial cells were compared by two-tailed Student's *t*-test, using GraphPad Prism v6.04 (GraphPad Software, La Jolla, CA).

### MicroRNA Reverse Transcription and Quantitative Real-Time Polymerase Chain Reaction

MicroRNA RT-qPCR was performed using the MystiCq MicroRNA Quantification System (Sigma-Aldrich Merck), according to the manufacturer's instructions. Briefly, a poly(A) tailing reaction was carried out with 500 ng of RNA, incubated 60 min at 37°C and 5 min at 70°C. First-strand cDNA synthesis reaction was performed using 9 μL of MystiCq microRNA cDNA Reaction Mix, 10 μL of Poly(A) Tailing Reagent, and 1 μL of ReadyScript Reverse Transcriptase, incubated 20 min at 42°C and 5 min at 85°C. The PCR was performed with 12.5 μL of MystiCq microRNA SYBR Green qPCR ReadyMix, 0.5 μL of MystiCq microRNA qPCR Assay Primer (10 μM), 0.5 μL of MystiCq Universal PCR Primer (10 μM), 5 μL of microRNA cDNA (10 ng) and 6.5 μL of nuclease-free water for each reaction. Kit information for each transcripts is presented in Supplementary Table 2. Two-step amplification consisted of: a pre-cycling hold at 95°C for 2; 40 cycles of denaturation for 5 s at 95°C; and annealing for 30 s at 60°C. A melting curve, representing a 1-s hold at every 0.5°C between 70 and 95°C, was generated to confirm that a single peak was produced for each primer set. The cycle threshold and statistics were the same as for total RNA. Small nucleolar RNA, C/D box 44 (SNORD44) and miR-191-5p were used as reference genes.

### Alignments and Feature Counts

Paired-end FASTQ files were combined from lane-level files for all control and infected samples and aligned to the human hg19 reference genome and to the *T. gondii* reference sequence (ToxoDB release 36, http://toxodb.org/toxo/showXmlDataContent.do?name=XmlQuestions.News&tag=toxodb02_18_release) using the subread-align program (31). Uniquely mapped reads were assigned to human and *T. gondii* genes with the read summarization program featureCounts (32). Reversely stranded read counting was performed.

### Filtering and Normalization

For subsequent analysis of the human genes, lowly expressed genes were removed; only genes which were expressed at a level of 2 counts per million in at least 2 samples were retained. Data normalization was performed using all samples to compare the ARPE-19 cell line with the primary retinal pigment epithelial cells and upper quartile normalization. For further analyses using only the primary cells, the ARPE-19 cell line was removed, and the data re-normalized with the conditional quantile normalization (CQN) method, which corrects for subject-specific bias and for systematic biases due to gene length and guanine-cytosine (GC) content (33). For this procedure, gene lengths and GC content were calculated from the hg19 GTF annotation file and FASTA file, respectively (http://ftp.ensembl.org/pub/). Multi-dimensional scaling plots were generated with limma to provide unsupervised clustering of samples (34). Pearson's Correlation was performed using the top 2,000 most variable genes to explore the relationships among the samples. The top 2,000 most variable genes were used because low variance among most genes will inflate correlation values. Normalization and all subsequent analyses were carried out using R version 3.2.2.

### Differential Expression Analysis

A paired statistical analysis was performed on the transcriptomes of the three paired primary retinal pigment epithelial cell samples, using the edgeR RNA-Seq expression analysis package (35), and with the entire dataset normalized together and a significance level of *p* < 0.05. These values were compared to expression values for the ARPE19 cell line. Subsequently, the CQN-computed offsets and fitting gene-wise negative binomial generalized log-linear models, likelihood ratio tests for infected vs. infected sample effect were conducted for the primary cells only. To correct for false discovery across multiple tests, *p*-values were adjusted using the Benjamini-Hochberg method. Genes having an FDR-adjusted *p*-value of less than 0.05 and fold change greater than 2 or lower than −2 were considered significantly differentially expressed. Ensembl IDs of the tested genes were mapped to approved HUGO Gene Nomenclature Committee (HGNC) symbols and descriptions obtained from the BioMart human Ensembl dataset (36).

### Gene Ontology and Pathway Enrichment

Ontology and pathway enrichment analysis was carried out with GOSeq statistical package (37). Gene Ontology biological process and molecular function categories (38) and Reactome pathways (39) were tested for over-representation in the set of differentially expressed genes, and *p*-values were adjusted for multiple testing using the Benjamini-Hochberg method. Categories were considered significant at an adjusted *p*-value of 0.05 or lower. To detect differential expression of sets of genes representing gene ontology categories and Reactome pathways, edgeR's implementation of CAMERA was used to perform competitive tests that account for inter-gene correlation (40); *p*-values were adjusted to account for multiple testing. Mappings between genes and Reactome pathway IDs were made with Entrez Gene IDs downloaded from BioMart.

### Network Analysis

To construct a network of molecular interactions involving differentially expressed genes and gene products, and their first-neighbor interactors (genes and gene products that interact directly with the differentially expressed genes), the Contextual Hub Analysis Tool (CHAT), a Cytoscape plugin, was used to query curated interactions in InnateDB, a public database of genes, proteins, experimentally-verified interactions, and signaling pathways involved in innate immune response (41, 42). Contextually relevant hubs, which interacted significantly with differentially expressed genes, were identified by CHAT. The network was visualized with Cytoscape v3.6 (43). The jActiveModules plugin identified subnetworks with high activity (44), using the false discovery rate from the differential expression analysis as the quantitative data associated with each differentially expressed gene (i.e., numeric node attribute), and with the parameters: 5 modules, overlap threshold 0.3, and search depth 1. The semantic similarity of the sets of enriched gene ontology categories associated with each of the 5 highly active subnetworks via CAMERA analysis was calculated with the Bioconductor GOSemSim package (45) using the Wang measurement method and the Best-Match Average (BMA) strategy for combining semantic similarity scores of multiple gene ontology terms. REVIGO (Reduce & Visualize Gene Ontology) and Treemap (46) were used to summarize and obtain representative terms, while removing redundant terms, for enriched gene ontology categories in the active subnetworks; an allowed similarity setting of 0.5 was used with the SimRel semantic similarity measure.

## Results

The response of human retinal pigment epithelial cells to infection with GT-1 strain *T. gondii* tachyzoites was interrogated by RNA-Seq of total and small RNA extracted from primary cells of three donors and the ARPE-19 cell line 24 h following infection. For total RNA, there were 87.7 × 10^6^ mean paired reads across the 8 samples: averaging across uninfected samples, 60.3% of reads aligned to the human genome, and 71.26% of reads were assigned to genes. For small RNA, there were 43.3 × 10^6^ mean reads across the 8 samples: averaging across uninfected samples, 0.23% of reads aligned to the human genome, and 0.66% of reads were assigned to genes. Alignment statistics are presented in Supplementary Table 3.

**Figure 1:**
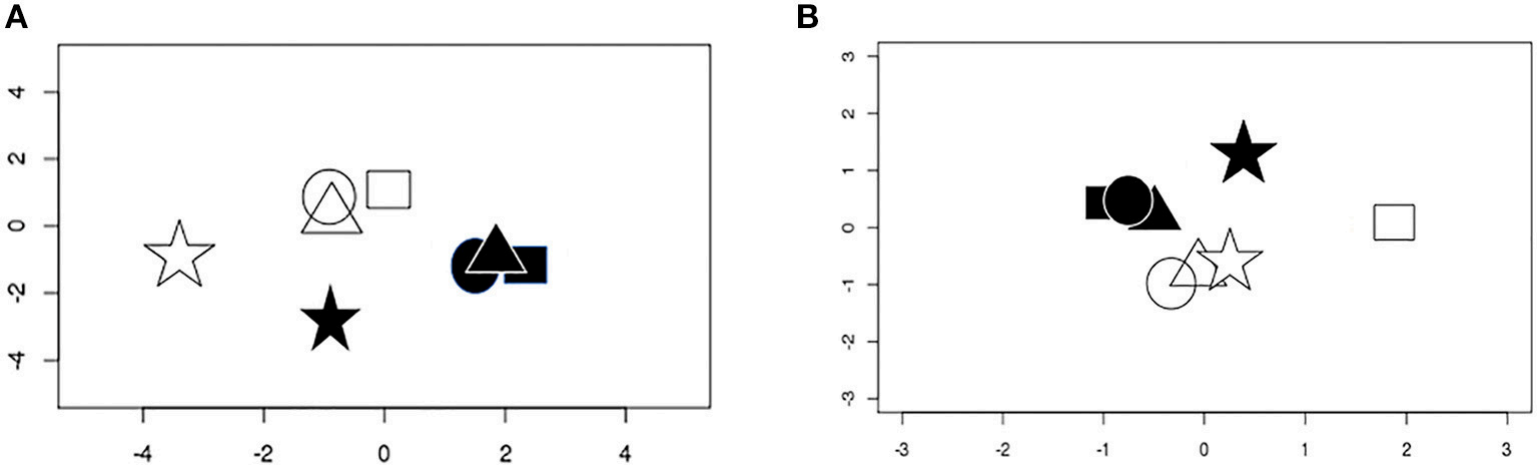
Multidimensional scaling plots of gene expression in uninfected and *T. gondii*-infected samples. **(A)** Conditional quantile normalized data from sequencing of total RNA. **(B)** Upper quartile normalized data from small RNA. Shapes represent different cell isolates: square, donor 1; circle, donor 2; triangle, donor 3; star, ARPE-19. Open symbols are uninfected samples and solid symbols are infected samples.

Multidimensional scaling demonstrated obvious separation between the total RNA transcriptomes of epithelial cells infected with *T. gondii* vs. those of uninfected cells (Figure 1A). However, while total RNA gene expression of the three primary cell isolates at baseline was similar and infection with *T. gondii* induced comparable changes, the ARPE-19 cell line had a different pattern of gene expression to the primary cells under control and infected conditions. Correlations among the top 2,000 most variable genes across all samples showed that expression of primary cells had 0.86–0.97 correlation, whereas ARPE-19 cell expression was correlated between 0.53 and 0.68 with that of primary cells (Supplementary Figure 1). On applying a gene expression threshold that was 3 standard deviations outside the gene expression of the three primary retinal epithelial cell isolates, 1,823 and 19,911 genes were more and less highly expressed, respectively, by the ARPE-19 cell line after infection with *T. gondii* (Supplementary Tables 4, 5). For small RNA, multidimensional scaling again showed separation between transcriptomes of *T. gondii*-infected and uninfected epithelial cells, but with more variation in global gene expression between the samples (Figure 1B); two primary cell isolates had similar gene expression under the two conditions, while the third primary cell isolate varied from this, and the ARPE-19 cell line again had a unique pattern.

**Figure 2:**
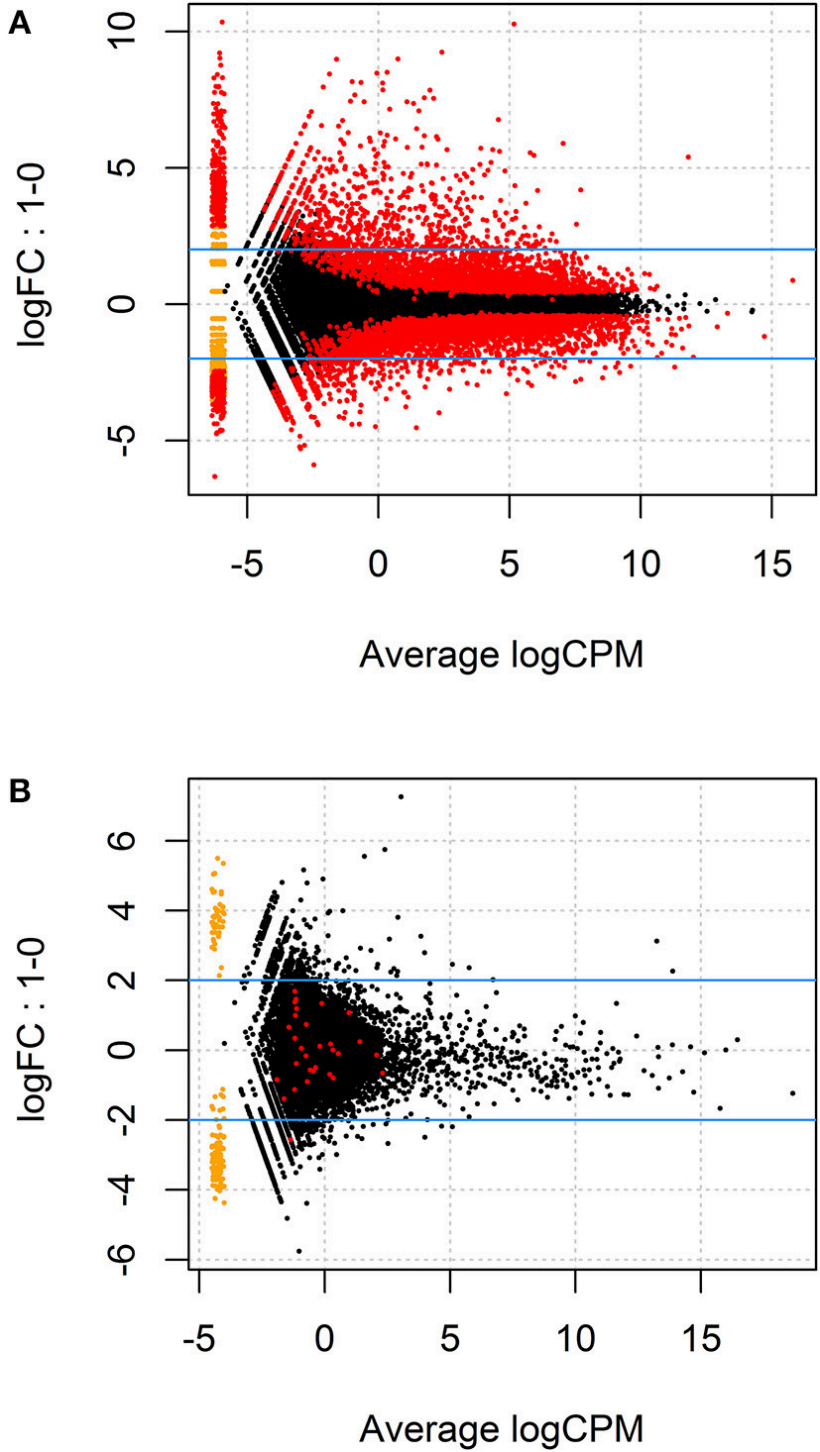
MA plots displaying differentially expressed genes for **(A)** total RNA and **(B)** small RNA between *T. gondii*-infected and uninfected human retinal pigment epithelial cells. The y-axis indicates log fold change observed in expression and the x-axis displays mean log of counts per million (CPM) of mapped reads (i.e., raw counts divided by total number of transcript regions sequenced multiplied by one million). The blue lines mark the (+) and (–) 2-fold change limits, and red dots above the upper blue line indicate the number of genes that were expressed >2-fold, and red dots below the lower blue line show genes that were expressed <2-fold, in uninfected vs. infected samples. The most highly significant changes appear on the far right of the x-axis below and above the 2-fold change limits.

**Figure 3:**
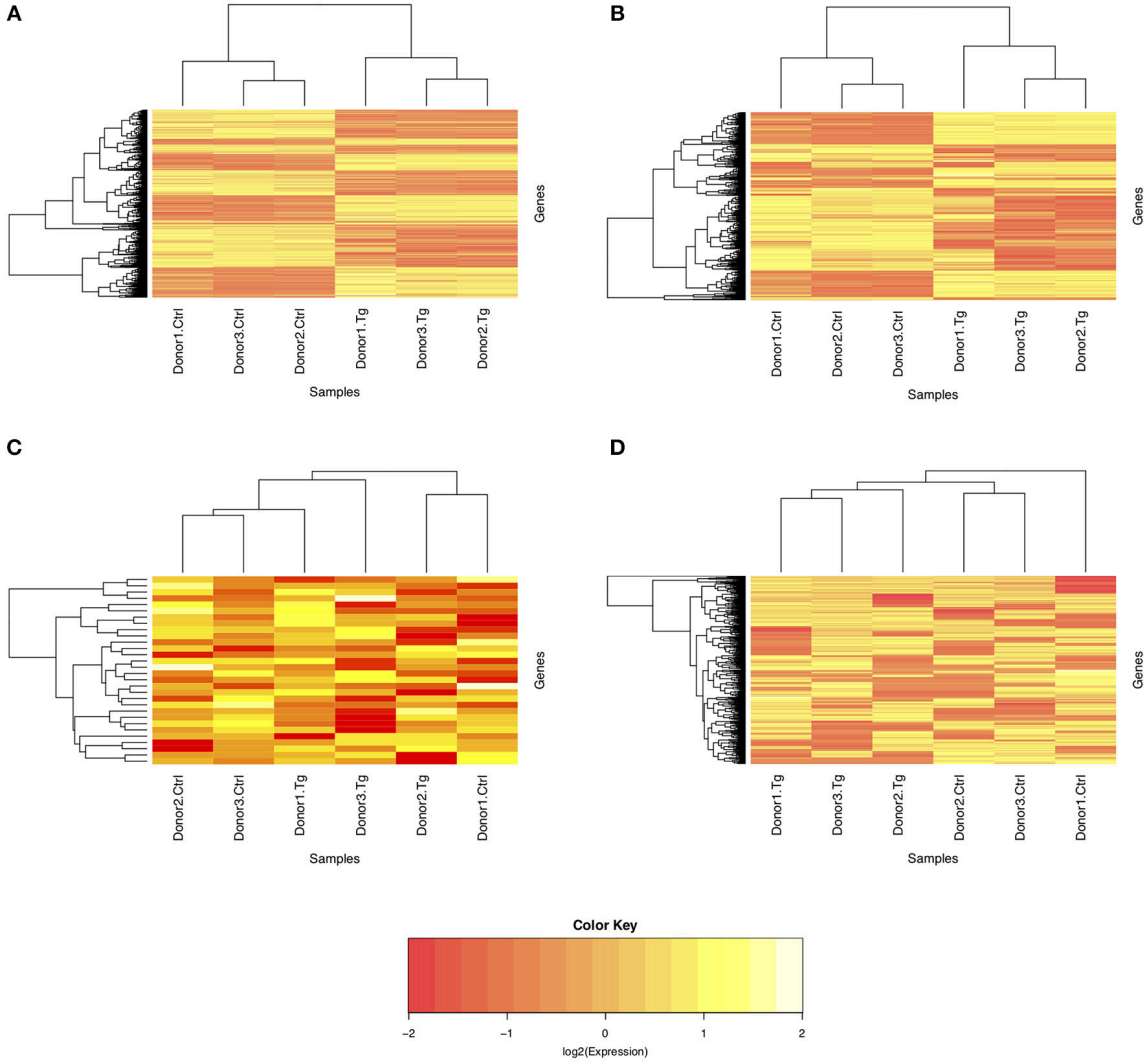
Heat maps of normalized counts per million from sequencing of total and small RNA. Expression on the log_2_ scale is shown for **(A)** all differentially expressed genes from total RNA, **(B)** the 1,000 most variable genes from total RNA, **(C)** all differentially expressed genes from small RNA, and **(D)** the 1,000 most variable genes from small RNA. The color scale changes from yellow to red, with greater intensity yellow indicating higher counts for transcripts in either uninfected control or *T. gondii*-infected cells from three donors.

Given the paired study design and clear separation of gene expression in *T. gondii*-infected and uninfected samples, RNA-Seq results for primary retinal pigment epithelial cell isolates were studied by paired differential expression analysis. For total RNA, 7,234 genes were significantly differentially expressed between infected and uninfected cells, including 1,969 that were up-regulated over 2-fold, and 1,739 that were down-regulated over 2-fold (Figure 2A and Supplementary Table 6), with heat-maps showing obvious clustering of differentially expressed transcripts (Figures 3A,B). In contrast, for small RNA, just 30 genes were significantly differentially expressed between infected and uninfected cells, including 26 that were up-regulated over 2-fold, and 4 that were down-regulated over 2-fold (Figure 2B and Supplementary Table 7), and there was no apparent clustering of differentially expressed transcripts (Figures 3C,D); 8 of the 30 significantly, highly differentially expressed genes were microRNA (miRNA).

**Figure 4:**
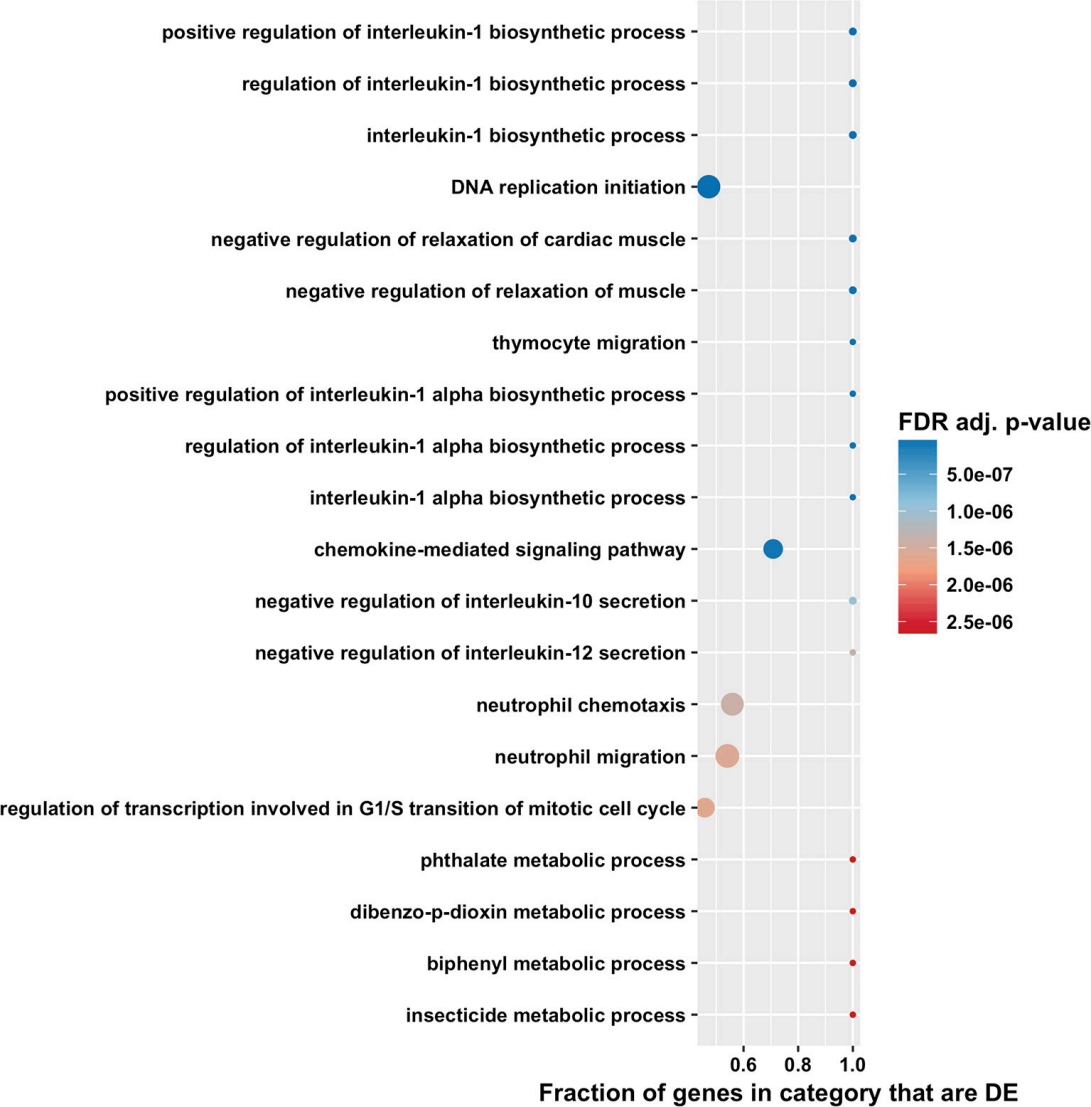
Gene ontology biological process enrichment analysis by CAMERA of differential expression in total RNA. Dotplots show the 20 most significantly enriched gene ontology biological process. Point size corresponds to the size of the gene ontology category, and points are plotted horizontally by the fraction of genes in the category that are differentially expressed. The color scale changes from red to blue, with blue indicating lower (more significant) adjusted *p*-value for the category.

**Figure 5:**
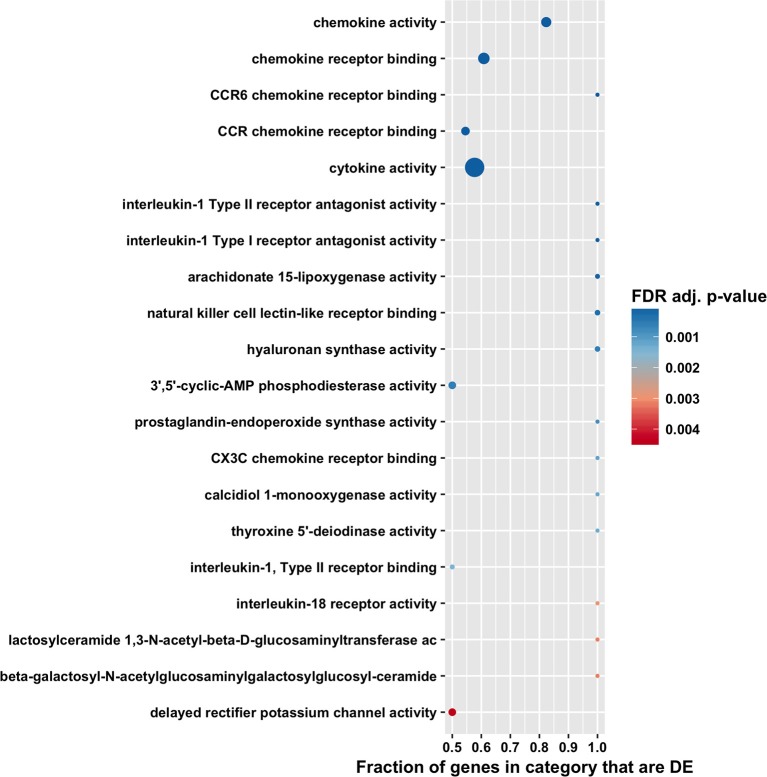
Gene ontology molecular function enrichment analysis by CAMERA of differential expression in total RNA. Dotplots show the 20 most significantly enriched gene ontology molecular function. Point size corresponds to the size of the gene ontology category, and points are plotted horizontally by the fraction of genes in the category that are differentially expressed. The color scale changes from red to blue, with blue indicating lower (more significant) adjusted *p*-value for the category.

**Figure 6:**
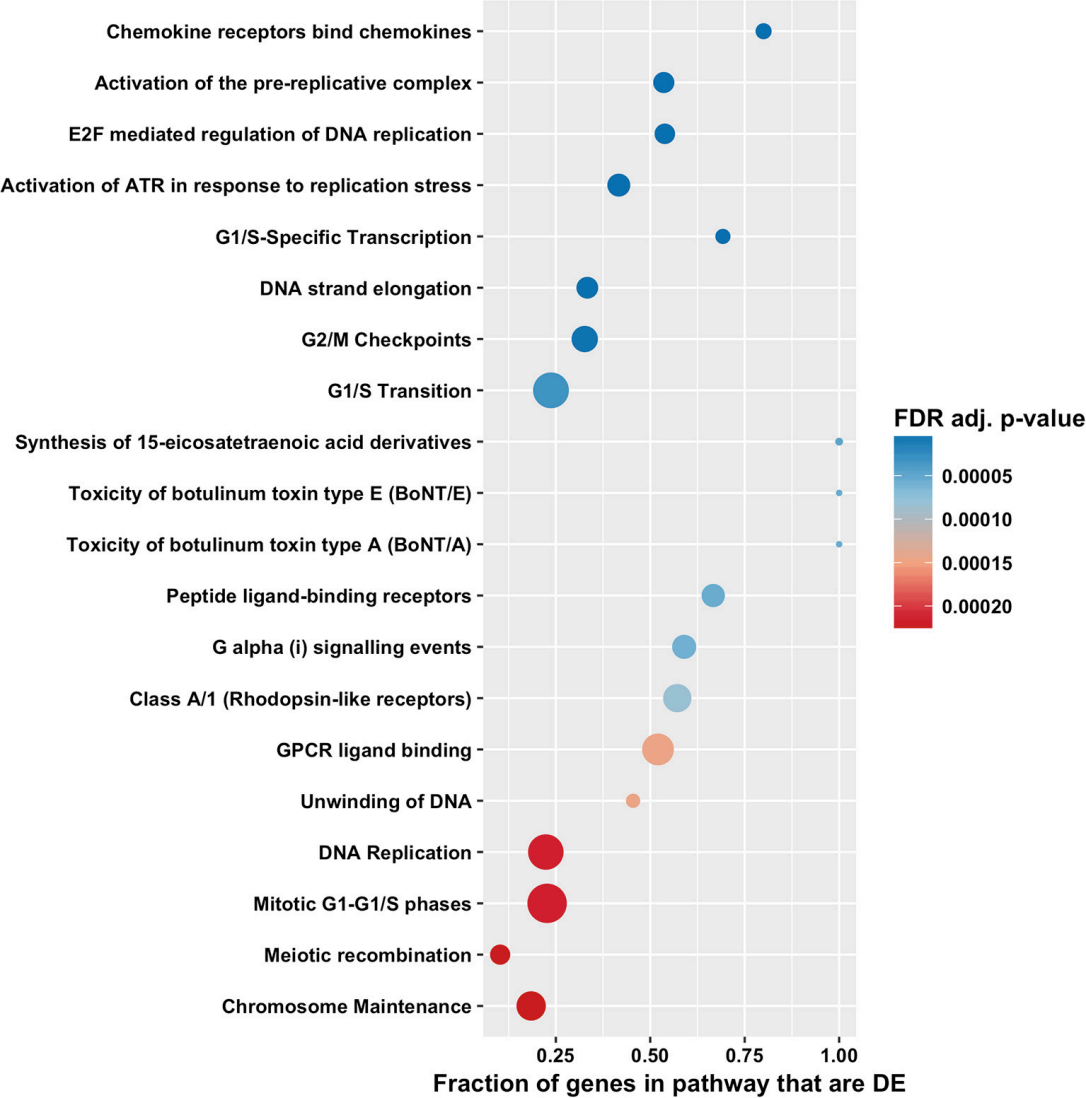
Pathway enrichment analysis by CAMERA of differential expression in total RNA. Dotplots show the 20 most significantly enriched Reactome pathways. Point size corresponds to the size of the Reactome pathway, and points are plotted horizontally by the fraction of genes in the category that are differentially expressed. The color scale changes from red to blue, with blue indicating lower (more significant) adjusted *p*-value for the category.

The high number of differentially expressed genes identified in the total RNA transcriptome indicated that infection with *T. gondii* resulted in massive disturbance of gene expression in the retinal pigment epithelial cell. Since this finding implied that expression of virtually all gene sets would be dysregulated in an infected epithelial cell, biological process and molecular function gene ontology, and Reactome pathway enrichment analyses were performed using the CAMERA software package, which accounts specifically for inter-gene correlation. The two gene ontology analyses indicated strong immunological signatures: 12 of the top 15 enriched biological process categories and 11 of the top 15 enriched molecular function categories were directly relevant to the immune response (Figures 4, 5, 6, and Supplementary Tables 8, 9). The list of top enriched pathways included immunological groupings, but also highlighted the activation of broadly applicable cell cycle and cell signaling processes.

**Figure 7:**
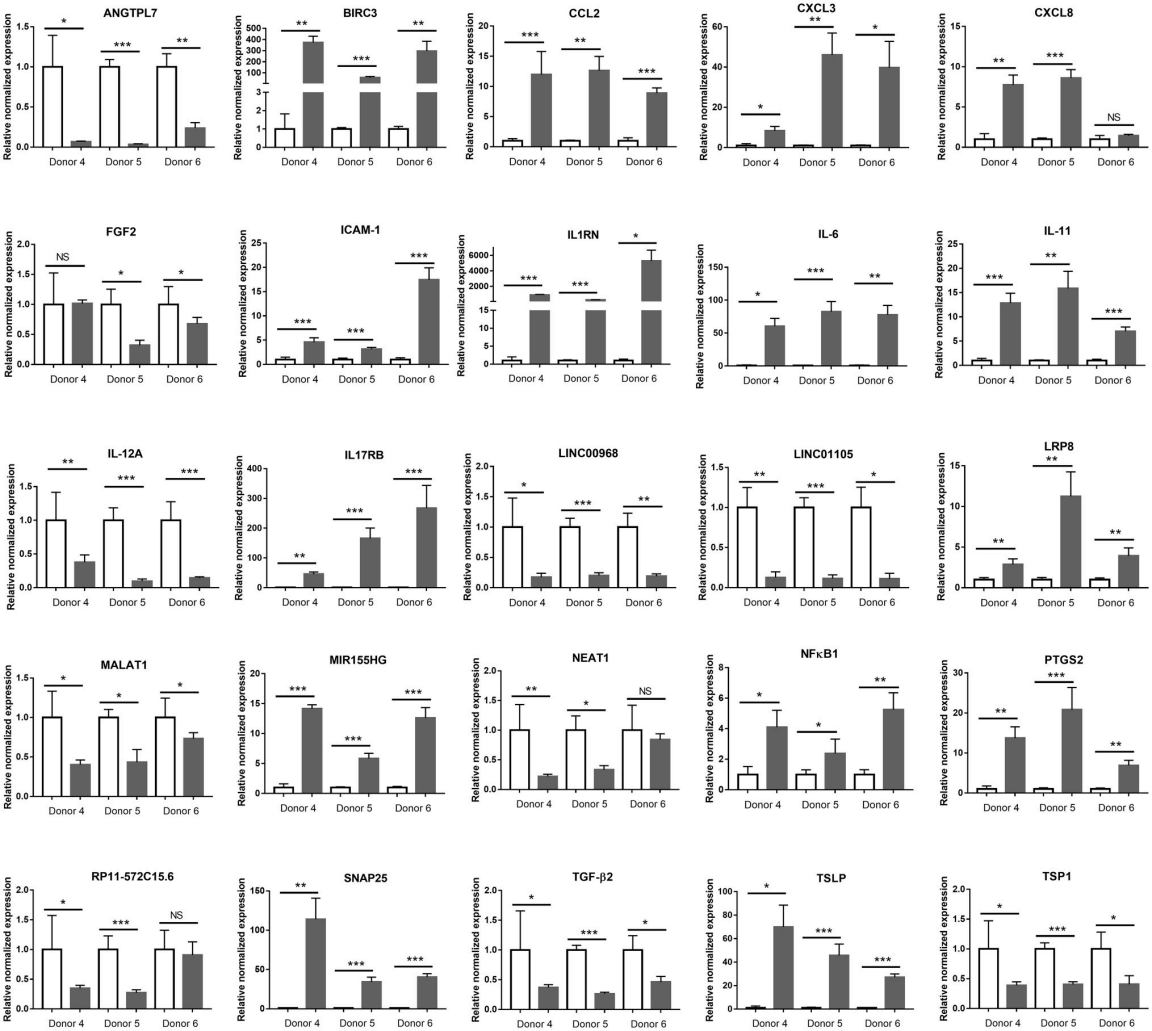
Graphs showing relative transcript expression for selected immune response molecules in *T. gondii*-infected primary human retinal epithelial cells vs. uninfected cells. Reference genes were ribosomal protein lateral stalk subunit P0 (RPLP0) and peptidylprolyl isomerase A (PPIA). Open bars represent uninfected cells and solid bars indicate *T. gondii*-infected cells. Bars represent mean relative expression, with error bars showing standard error of the mean. *n* = 4 cultures/donor and condition. Data were analyzed by two-tailed Student's *t*-test. ^*^p < 0.05, ^**^p < 0.01; ^***^p < 0.001.

To validate the immunological signature for the interaction between retinal pigment epithelium and *T. gondii* that had been identified by *in silico* analysis of the RNA-Seq data, changes in 20 protein-coding and 6 long non-coding immune response-related transcripts were studied by RT-qPCR on primary epithelial cell isolates from three additional donors. Sixteen transcripts were significantly increased, and 10 transcripts were significantly decreased in the RNA-Seq study. Of these 26 molecules, the same expression changes were observed in: three of 3 donors for 20 molecules (i.e., angiopoietin-like 7 [ANGTPL], baculoviral IAP repeat containing 3 [BIRC3], CCL2, CXCL3, intercellular adhesion molecule-1 [ICAM-1], interleukin-1 receptor antagonist [IL1RN], IL-6, IL-11, long intergenic non-protein coding RNA [LINC] 00968, LINC01105, low-density lipoprotein receptor-related protein 8 [LRP8], metastasis associated lung adenocarcinoma transcript 1 [MALAT1], miR-155 host gene [MIR155HG], nuclear factor kappa B subunit 1 [NFκB1], prostaglandin-endoperoxide synthase 2 [PTGS2], synaptosomal-associated protein 25 [SNAP25], transforming growth factor β2 [TGF-β2], thymic stromal lymphopoietin [TSLP], and thrombospondin 1 [TSP1]); in two of 3 donors for 4 molecules (i.e., CXCL8, fibroblast growth factor 2 [FGF2], nuclear paraspeckle assembly transcript 1 [NEAT1] and RP11-572C15.6); and in one of 3 donors for one molecule (i.e., vascular cell adhesion molecule 1 [VCAM-1]) (Figure 7 and Supplementary Table 10).

**Figure 8:**
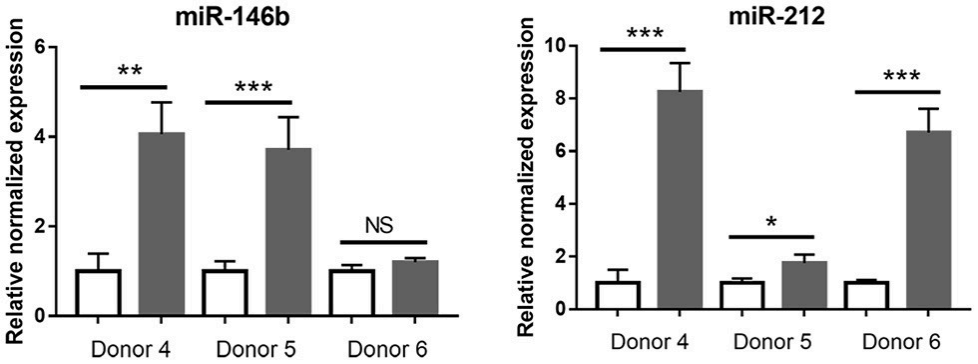
Graphs showing relative transcript expression for selected differentially expressed microRNA in *T. gondii*-infected primary human retinal epithelial cells vs. uninfected cells. Reference genes were small nucleolar RNA, C/D box 44 (SNORD44), and miR-191-5p. Open bars represent uninfected cells and solid bars indicate *T. gondii*-infected cells. Bars represent mean relative expression, with error bars showing standard error of the mean. *n* = 4 cultures/donor and condition. Data were analyzed by two-tailed Student's *t*-test. ^*^p < 0.05, ^**^p < 0.01; ^***^p < 0.001.

Given the low number of significantly changed small RNA in *T. gondii*-infected retinal pigment epithelial cells, that data set was not tested by enrichment analysis. However, RT-qPCR was used to examine the response to infection of seven of the 8 differentially expressed miRNA identified by RNA-Seq, in the cell isolates from the three additional donors. Of these 7 miRNA, the same expression changes were observed in: three of 3 donors for one miRNA (i.e., miR-212); two of 3 donors for one miRNA (i.e., miR-146B); one of 3 donors for three miRNA (i.e., miR-449C, miR-670, and miR-3130-1); and none of 3 donors for 2 miRNA (i.e., miR-10B and miR-449B) (Figure 8 and Supplementary Table 11). Both miR-212 and miR-146B have been implicated in regulation of the immune response (47).

**Figure 9:**
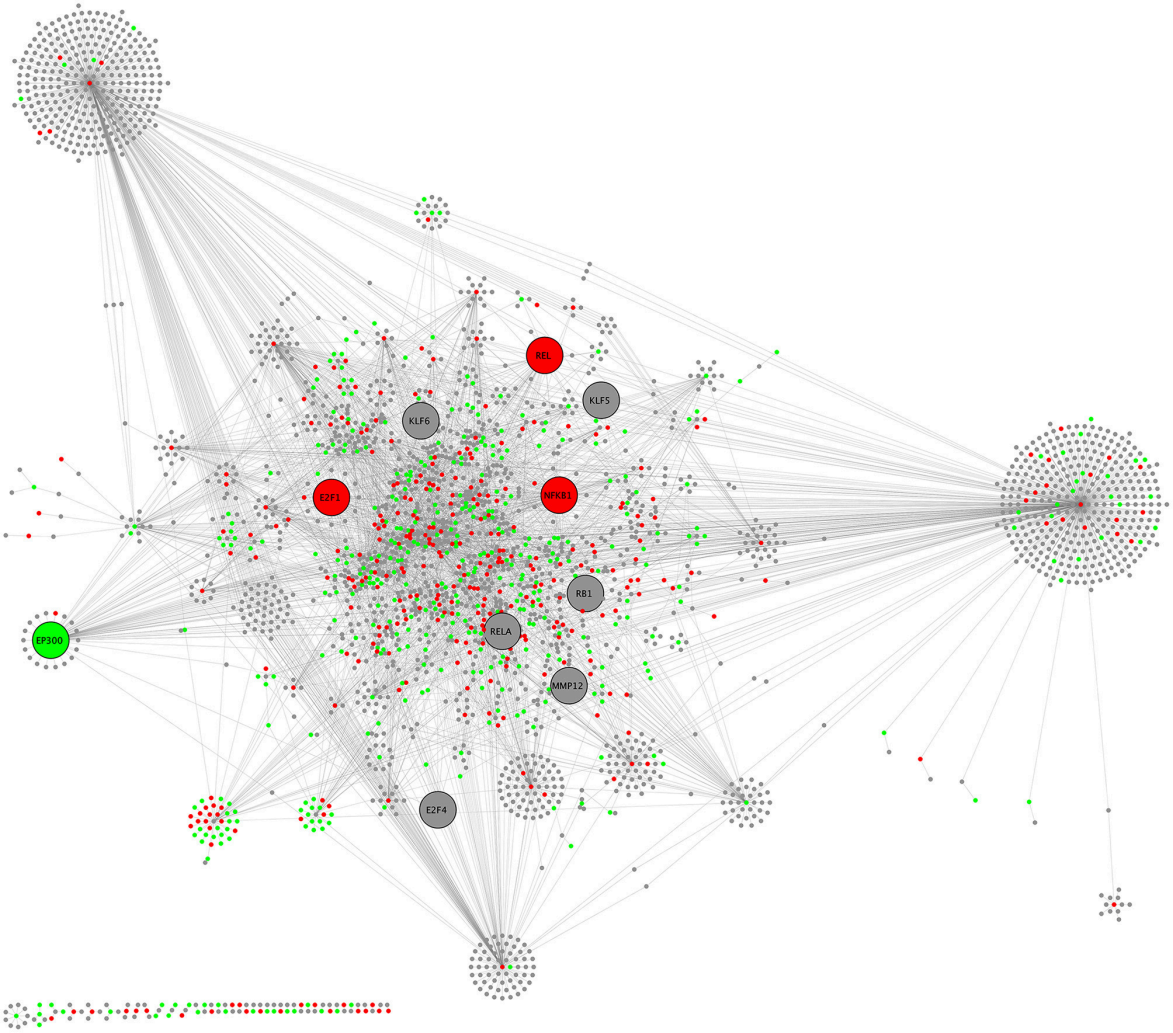
CHAT network schematic of interactions between differentially expressed genes by sequencing of total RNA, as annotated in InnateDB. Lines represent molecular interactions; red nodes are upregulated transcripts; green nodes are downregulated transcripts; and gray nodes are first-neighbor interactors that are not differentially expressed. Large nodes indicate contextual hubs.

**Figure 10:**
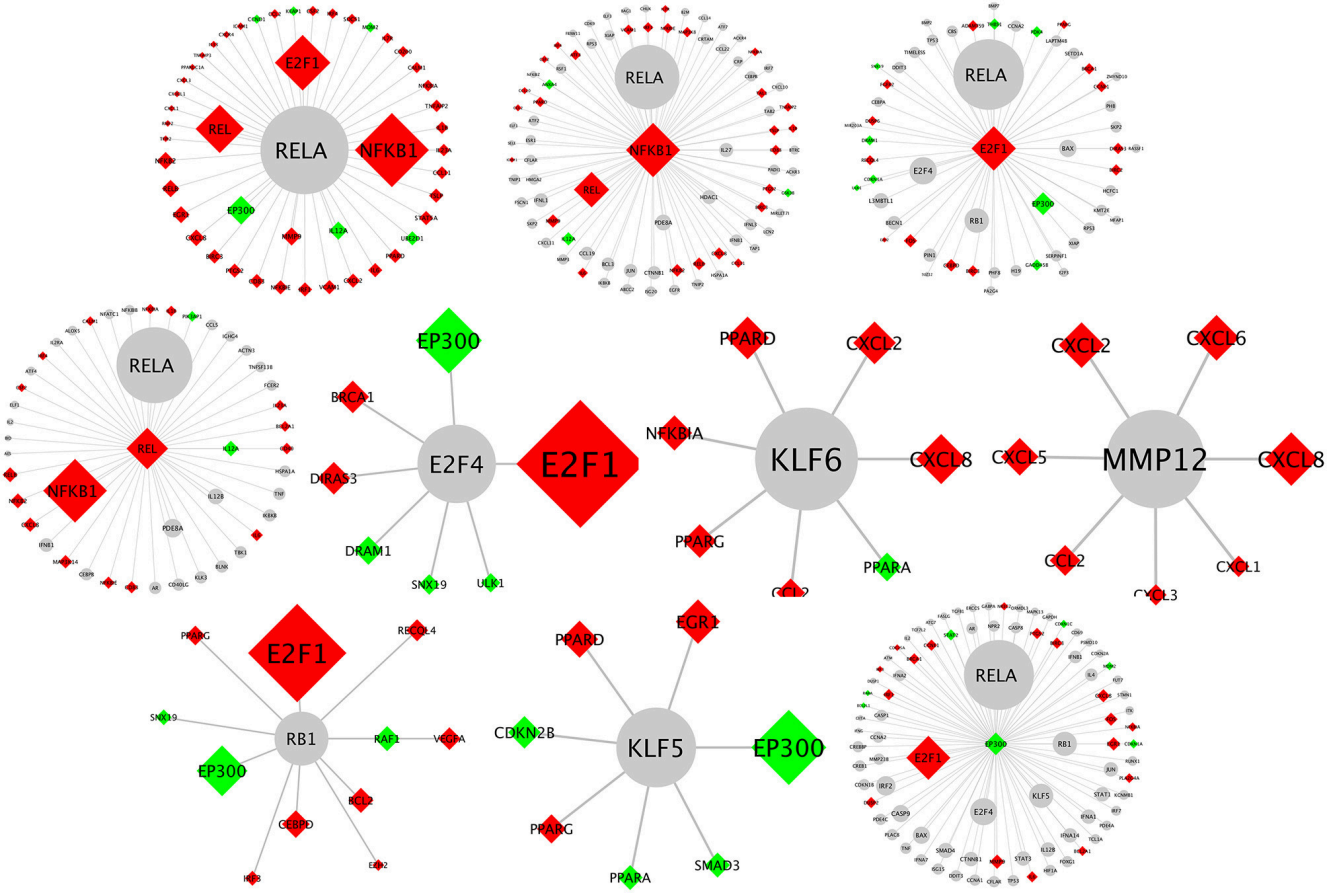
Network schematics illustrating the first neighbor interactors of each of the contextual hub genes listed in Table 1. The contextual hub gene is at the center of each network diagram. Lines represent molecular interactions; red diamonds indicate upregulated transcripts; green diamonds indicate downregulated transcripts; and gray nodes are interactors that are not differentially expressed. Larger nodes correspond to more statistically significant hubs as calculated by the CHAT application.

**Figure 11:**
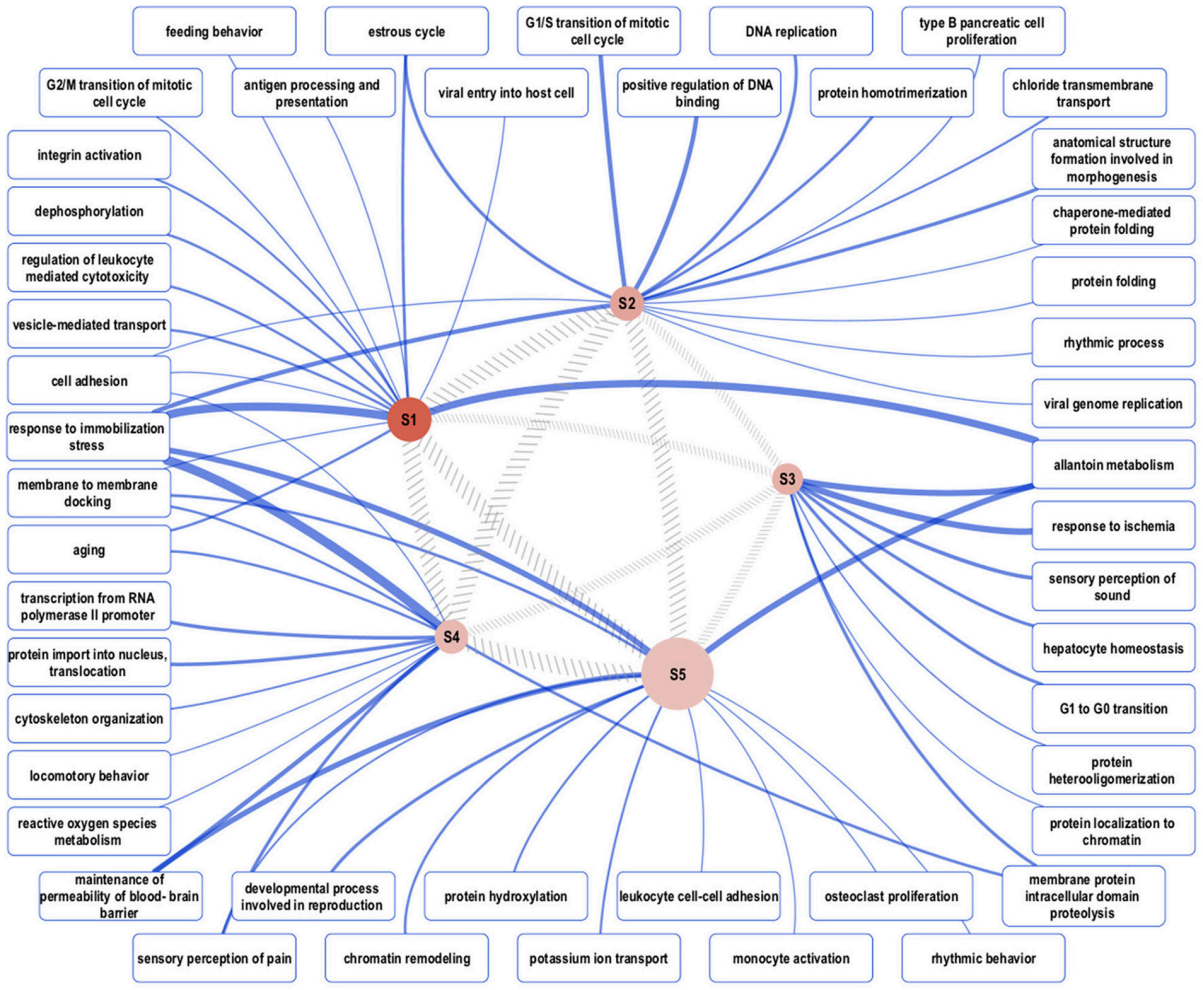
Overview of the representative gene ontology biological process categories that are associated with each of five subnetworks with high activity. Semantically representative categories were identified by REVIGO Treemap analysis of all enriched categories in each subnetwork. The reddish-brown nodes, labeled S1–S5, represent the subnetworks, and their size is proportional to the total number of genes in the subnetwork. Darker shading corresponds to greater pathway activity. The width of the hatched gray lines between the subnetwork nodes is proportional to the semantic similarity between each, based on enriched categories. The widths of the blue lines connecting representative gene ontology categories to the subnetworks correspond to the total number of categories that map to the representative category.

**Figure 12:**
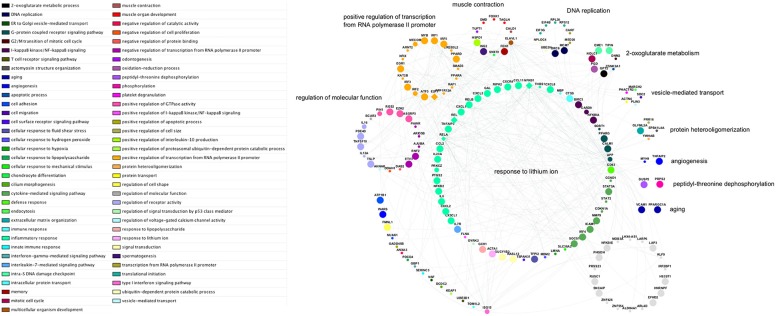
Network schematic of genes, interactions, and gene ontology biological process groupings in the subnetwork with highest activity. Genes are grouped according to the semantic summarization of the most significantly enriched category—represented by colored nodes—associated with each gene; the groups are labeled with representative terms identified by REVIGO Treemap analysis. Grey nodes represent genes that are not associated with any of the enriched categories for the subnetwork. Large nodes indicate upregulated transcripts; small nodes indicate downregulated transcripts; and medium-sized nodes indicate first neighbor interactors that are not differentially expressed. Diamond-shaped nodes indicate contextual hub genes. Each of the genes within the arc at lower-left is associated with a gene ontology category that is not included in any larger grouping by the Treemap analysis.

Biological network analysis was performed in InnateDB, which is a manually curated, systems immunology platform: 735 molecules encoded by differentially expressed transcripts were annotated in InnateDB, and an additional annotated 2046 first-order interactors were identified, giving a total of 2781 molecules. This analysis identified an extensive molecular network (Figure 9 and Supplementary Table 12) with 10 highly connected nodes, known as contextual hubs (Table 1 and Figure 10), and 5 subnetworks or modules with high levels of activity (Figure 11). These subnetworks presented distinct and overlapping processes, multiple of which would be expected to regulate leukocyte behavior, including adhesion, activation and effector functions. Biological process gene ontology enrichment analysis of the most active subnetwork elaborated on the observation, by identifying specific immune response-related processes, including multiple cytokine-mediated processes, signaling pathways and danger-associated responses (Figure 12 and Supplementary Table 13).

**Table 1:** Contextural hubs expressed by primary human retinal pigment epithelial cells 24 h following infection with *T. gondii*.

**Symbol**	**Name**	**Contextual neighbors**	**Total neighbors**	**False discovery rate**
RELA	RELA proto-oncogene, NF-KB subunit	47	154	0.00001
NFKB1	Nuclear factor kappa B subunit 1	30	84	0.00008
E2F1	E2F transcription factor 1	21	54	0.001
REL	REL proto-oncogene, NF-KB subunit	18	45	0.003
E2F4	E2F transcription factor 4	7	10	0.022
KLF6	Kruppel like factor 6	7	10	0.022
MMP12	Matrix metallopeptidase 12	7	11	0.036
RB1	RB transcriptional corepressor 1	11	25	0.036
KLF5	Kruppel like factor 5	7	11	0.036
EP300	E1A binding protein P300	24	87	0.046

## Discussion

Ocular toxoplasmosis is a common, vision-threatening eye disease that is characterized clinically and histopathologically by disruption of the retinal pigment epithelium (4). Multiple studies have described specific molecular interactions between the human retinal pigment epithelial cell and the rapidly proliferating tachyzoite form of the parasite (14–18). We present a transcriptomic view of the epithelial cell response to infection with *T. gondii*. RNA-Sequencing of total and small RNA extracted from three human primary retinal pigment epithelial isolates infected with virulent, natural GT-1 strain tachyzoites enabled *in silico* differential expression, and gene ontology, pathway and network enrichment analyses. Our work revealed a sizeable molecular response in the total RNA transcriptome—with significantly differentially expressed genes totaling 28.9% of total assigned transcripts—but very limited consistent changes in the small RNA transcriptome—totaling 0.35% of assigned transcripts. Gene ontology and pathway studies of differentially expressed total RNA identified a strong immune response signature, which we validated by RT-qPCR for multiple immune response-related protein-coding and long non-coding transcripts in human retinal pigment epithelial cells isolated from three independent donors. By performing biological network analyses within the InnateDB systems immunology platform we identified contextural hubs and subnetworks that illuminated the global immune response to the parasite.

The human retinal pigment epithelial cell total RNA transcriptome was substantially impacted by infection with *T. gondii*: approximately one-third of transcripts were significantly up- or down-regulated, with over 10% of transcripts having greater than two-fold change. *T. gondii* may infect any nucleated cells, but *in vitro* comparisons of global gene expression changes between heterogeneous cell populations (i.e., mouse skeletal muscle cells, neurons, astrocytes and fibroblasts), as well as related cell subsets (i.e., human dendritic cells and macrophages), suggest substantial differences in the extent, as well as spectrum, to which transcription is impacted within the first 24 h of infection (48, 49). In a previous gene expression microarray profiling study from our group, less than 1% of transcripts changed in human retinal endothelial cells similarly infected with *T. gondii*, albeit for a shorter time interval (50). A high proportion of gene expression changes might reflect disruption of the diverse functions of the retinal pigment epithelial cell and/or a more specific vigorous immunologic reaction to *T. gondii* infection. While the infection is expected to elicit some degree of immune response in most cell populations, the retinal pigment epithelium in particular is likely to respond vigorously, being a key contributor to ocular immune privilege in the posterior eye (51). Indeed, enrichment analyses identified high immune response-related activity in these cells.

Ocular immune privilege describes the tissue, cellular and molecular mechanisms that limit inflammation inside the eye, to protect ocular immune functioning for preservation of vision (52). The retinal pigment epithelium presents a physical barrier, and expresses an array of molecules that act to downregulate the activity of innate and adaptive immune cells (51). Overall, however, infection with *T. gondii* appeared to push the epithelial cell toward a molecular phenotype that might promote inflammation. This breach of immune privilege is seen in other ocular infections and often is believed to be the primary cause of tissue destruction in the disease (53). Gene ontology and pathway analyses of the RNA-Seq data identified activity in chemokine, cytokine and arachidonic acid-related categories, and a majority of contextual hubs identified in the network analysis were transcriptional regulators linked to inflammation, including members of the NF-kB family. At the molecular level, both RNA-Seq and RT-qPCR revealed that changes in levels of immunomodulatory molecules varied: for example, increase in the level of IL1RN, which potently inhibits the activity of IL-1β (54), was counterbalanced by decrease in the level of the key intraocular immunosuppressive factor, TGF-β2, as well as TSP1, which activates TGF-β2 (55). In parallel, expression of an array of pro-inflammatory cytokines and other molecules was increased. Yet, infected retinal pigment epithelial cells negatively regulated IL-12 production, which acts as the key link in innate and adaptive immune responses against *T. gondii*, by triggering a Th1 response that involves multiple intracellular mechanisms targeted against the parasite (56).

In contrast to the substantial changes in retinal pigment epithelial cell protein coding and long non-coding RNA, less than 1% of small RNA were differentially expressed by retinal pigment epithelial cells following *T. gondii* infection. These small RNA included just 8 miRNA, of which only two miRNA could be validated by RT-qPCR in epithelial cells from additional human donors. The markedly limited proportion of differential expression and low validation success may relate to level and stability of expression level, as well as inter-individual variation in the expression of these RNA. Interestingly, both validated miRNA—miR-146b and miR-212—have important roles in the regulation of immune responses (47). For example: miR-212 has been implicated in differentiation and activation of various lymphocyte subsets, including Th17 cells, B cells and NK cells, as well as macrophages (57–60); and miR-146b reduces inflammatory cytokine production by mature dendritic cells, and limits expression of adaptor molecules involved in toll-like and cytokine receptor signaling in macrophages (61, 62). MicroRNA-146 may be particularly relevant in the response of the retinal pigment epithelium to an intra-ocular pathogen: up-regulation of miR-146 is characteristic of infection with *Mycobacterium tuberculosis* (63), and like *T. gondii, M. tuberculosis* targets the retinal pigment epithelium inside the eye (64). MiR-146b interactors were well represented in the network analysis: three contextual hubs are directly involved in the NFkB pathway, which regulates and is regulated by miR-146b (62, 65); and miR-146b also participates in IL-1β signaling (66, 67), which was highlighted in the enrichment analyses.

Multidimensional scaling of the total RNA transcriptome indicated that global changes in protein coding and long non-coding RNA levels in infected primary human retinal pigment epithelial cells were consistent across donors, but varied from those in infected ARPE-19 cells. The ARPE-19 cell line was first reported in 1996, as a human cell line arising spontaneously from the culture of retinal pigment epithelial cells of a teenaged male cadaveric donor (21). We and others have used this line for the study of different retinal infections, including ocular toxoplasmosis (16–18, 68–71). While culture conditions were not identical for primary cell isolates and the cell line, the diversity of gene expression we observed is likely to reflect real phenotypic differences. The ARPE-19 line cell exhibits reduced expression of approximately 20% of a set of 154 epithelial cell signature genes, identified by transcriptomic profiling of multiple human native and cultured primary fetal and adult retinal pigment epithelial cell isolates (72). Proteomic comparison with primary adult cells indicates ARPE-19 cells express relatively high levels of cytoskeletal and proliferation-related proteins (73). Although not the primary focus on our work, we compared genes expressed by the ARPE-19 cell line and primary human retinal pigment epithelial cells after infection with *T. gondii* by gene ontology: the top 15 biological process categories for genes that were more highly expressed in ARPE-19 cells included 6 categories that were directly relevant to the immune response (Supplementary Tables 14, 15). This indicates the ARPE-19 cell line mounts an immunological reaction to the infection, but this response involves some different molecules to those involved in the response of primary cells. Given the origin of the cell, and the vast literature behind its use in ophthalmology research, the ARPE-19 cell line will almost certainly continue to be a valuable tool for investigating the role of retinal pigment epithelial cells in the human retinal disease. However, our results indicate that in studies of ocular toxoplasmosis, experimental findings generated with this line should be confirmed in primary cells. Even primary retinal pigment epithelial cells show phenotypic variation, depending on culture conditions (74), and we used two different isolation protocols to address this issue in validating the immune signature of infection.

In summary, this work sheds light on the RNA transcriptome of human retinal pigment epithelial cells following infection with *T. gondii*, and identifies a strong immunological gene expression response of these cells to the parasite. The experimental approach is a strength of our study, including validation of the immunological signature generated through RNA-Seq and *in silico* analyses, by RT-qPCR, and use of multiple primary human retinal pigment epithelial cell isolates. Separate groups of isolates were prepared according to different protocols for RNA-Seq and RT-qPCR. A limitation of our study is the focus on one time point post-infection, although 24 h is the central time for host response to parasite replication (28). We also focused on one virulent parasite strain: yet, *T. gondii* has a well described clonal population structure across the world, including standard or typical, and exotic or atypical strains (75). Virulent and avirulent parasite strains have been defined on the basis of behavior *in vivo* and *in vitro* experiments, and classified genetically (25, 26, 76); both virulent and avirulent strains may cause ocular toxoplasmosis in healthy adults (77–79). Despite these limitations, the data we present provide a solid base for future studies of molecular and cellular interactions between *T. gondii* and the retinal pigment epithelium to elucidate basic mechanisms of ocular toxoplasmosis. Future studies may compare the human retinal pigment epithelial cell transcriptomic response to infection with a virulent *T. gondii* strain, to transcriptomes of cells infected with avirulent strains and/or exotic strains, to better understand the full spectrum of ocular toxoplasmosis as it occurs across the globe.

## Author Contributions

SL, ER, BW, and JS conceived the study. SL, ER, AS, TB, MM, BA, BW, and JS designed the experiments. SL, ER, YM, and LA generated experimental data. ER, ES, MM, BA, BM, and JS analyzed and/or interpreted experimental data. ER, ES, BW, and JS drafted the paper. SL, YM, LA, AS, TB, MM, and BA provided critical review of the paper. All authors give approval for publication of the content.

### Conflict of Interest Statement

The authors declare that the research was conducted in the absence of any commercial or financial relationships that could be construed as a potential conflict of interest.
