# Supplementary material for: Immunological Molecular Responses of Human Retinal Pigment Epithelial Cells to Infection With Toxoplasma gondii
Source: Front Immunol. 2019 May 1;10:708. doi: 10.3389/fimmu.2019.00708 (PMC6506780; doi:10.3389/fimmu.2019.00708)
Supplement: Supplementary file 11 [file Data_Sheet_1.PDF]

Supplementary Material

Immunological Molecular Responses of Human Retinal Pigment Epithelial Cells to Infection with *Toxoplasma gondii*

Shervi Lie, Elise Rochet, Erik Segerdell, Yuefang Ma, Liam M. Ashander, Audra M. Shadforth, Timothy A. Blenkinsop, Michael Z. Michael, Binoy Appukuttan, Beth Wilmot, Justine R. Smith*

***Correspondence:** Justine Smith: justine.smith@flinders.edu.au

Supplementary Figure 1, and Tables S1-S3, S10 and S11 are included in this document. Supplementary Tables S4-S9 and S12-S15 are presented as separate .xlsx files.

Supplementary Figure S1. Correlogram comparing gene expression in uninfected and *T. gondii* infected samples for the top 2000 most variable genes. D = donor; 19 = ARPE19; Ctrl = control; Tg = *T. gondii*.

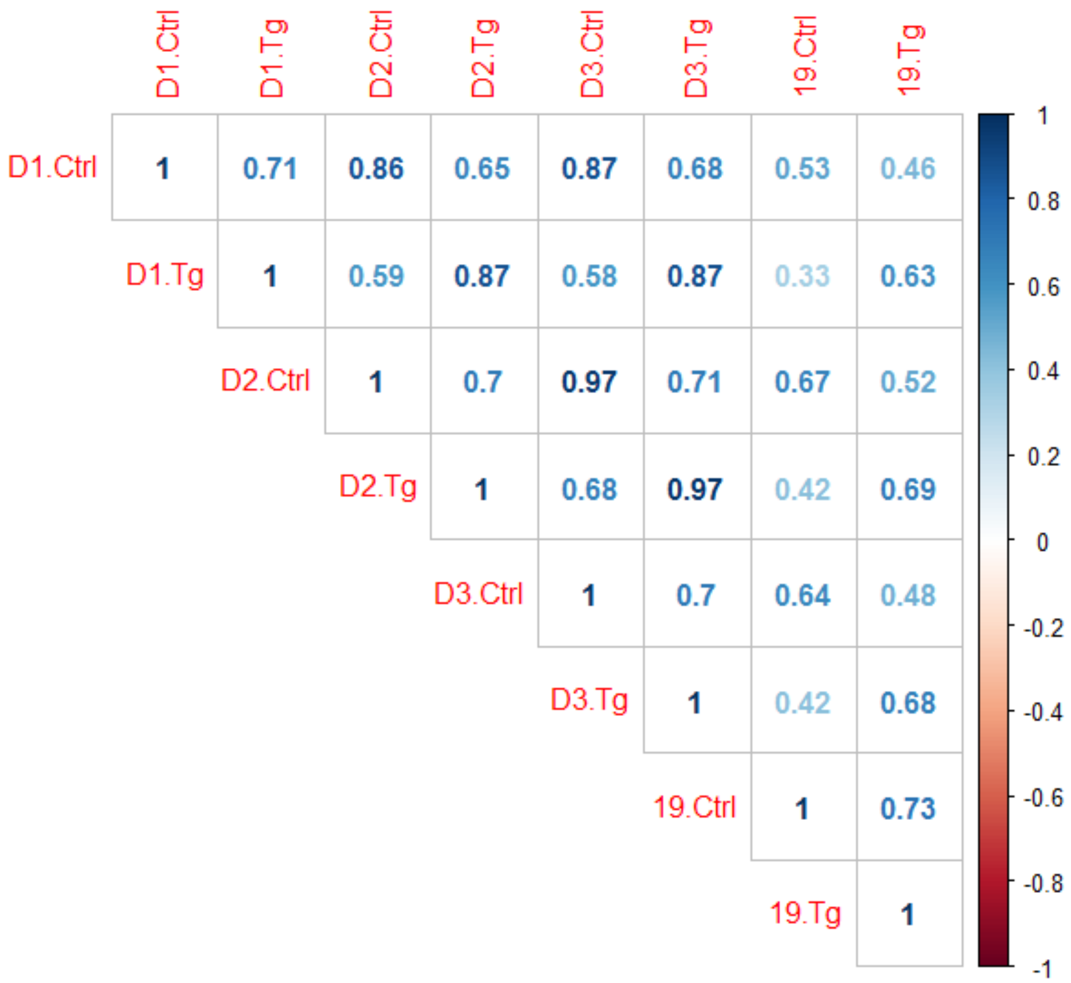

Supplementary Table S1. Primer pairs and product sizes for protein-coding and long non-coding transcripts.

Transcript*	Primer pair	Product size (bp)
ANGTPL7	Forward 5’- TGGAAGCAGTACAAGCAGGG -3’<br>Reverse 5’- GCTATACTCAGCGTAGCGCA -3’	147
BIRC3	Forward 5’- AGCAAAGCCATGCACAAAACT -3’<br>Reverse 5’- ACGTGTTGGCGCTTTTCATC -3’	127
CCL2[1]	Forward 5’- AATCAATGCCCCAGTCACCTGC -3’<br>Reverse 5’- CGGAGTTTGGGTTTGCTTGTCC -3’	211
CXCL3[2]	Forward 5’- TCATCAAACATAGCTCAGTCCTG -3’<br>Reverse 5’- GGCTGACACATTATG GTCTCC -3’	100
CXCL8[2]	Forward 5’- GTGTGAAGGTGCAGTTTTGC -3’<br>Reverse 5’- TCTGCACCCAGTTTTCCTTG -3’	203
FGF2	Forward 5’- CAAGCAGAAGAGAGAGGAGTTG -3’<br>Reverse 5’- GCTCTTAGCAGACATTGGAAGA -3’	273
ICAM-1[4]	Forward 5’- TAAGCCAAGAGGAAGGAGCA -3’<br>Reverse 5’- CATATCATCAAGGGTTGGGG -3’	289
IL1RN[2]	Forward 5’- TCATGCTCTGTTCTTGGGAAT -3’<br>Reverse 5’- GCTTGTCCTGCTTTCTGTTC -3’	131
IL-6[3]	Forward 5’- ATGAACTCC TTC TCCACAAGCGC -3’<br>Reverse 5’- GAAGAGCCCTCAGGCTGGACTG -3’	628
IL-11	Forward 5’ -GTCCCGGATTCTTGGGTCTC -3’<br>Reverse 5’- AAACCCAGGCTTCCCTTTCC -3’	151
IL-12A	Forward 5’- GTGGAGGCCTGTTTACCATT -3’<br>Reverse 5’- CGGTTCTTCAAGGGAGGATTT -3’	315
IL17RB	Forward 5’- GGGACCTCCGAGTAGAACCT -3’<br>Reverse 5’- CTTGGTGGCCTTCAACAAGC -3’	119
LINC00968	Forward 5’- AATTGTGTCCCCTGTCCACC -3’<br>Reverse 5’- CTGTGCTGAGCTGTCTGGAA -3’	126
LINC01105	Forward 5’- AGGAGAAGGAATTGTGGCGG -3’<br>Reverse 5’- CAGTCCCTGCTGTAGCG AA -3’	137
LRP8	Forward 5’- AAGTGTGTACCTGCCTCGTG -3’<br>Reverse 5’- CCGTCACCACAGTCGTCG -3’	167
MALAT1	Forward 5’- ATTCCGGTGATGCGAGTTGT -3’<br>Reverse 5’- ATTCGGGGCTCTGTAGTCCT -3’	396
MIR155HG	Forward 5’- CTTGGCTCTCCCACCCAATG -3’<br>Reverse 5’- CCTACAGCAAGCCTTCAGCA -3’	115
NEAT1	Forward 5’- CTTGGCACTGGTACTGGGAG -3’<br>Reverse 5’- ACCCACGCACTAAATTCCCC -3’	137
NFκB1[5]	Forward 5’- CCCAGTGAAGACCACCTCTC -3’<br>Reverse 5’- CTGAGTTTGCGGAAGGATGT -3’	132
PPIA	Forward 5’- GAGCACTGGAGAGAAAGGATT T -3’<br>Reverse 5’- GGTGATCTTCTTGCTGGTCTT -3’	355

PTGS2	Forward 5'- CCAGAGCAGGCAGATGAAATA -3'<br>Reverse 5'- CCAGTAGGCAGGAGAACATATAAG -3'	277
RP11-572C15.6	Forward 5'- ATCAGACATCTCCCAGGGCT -3'<br>Reverse 5'- ATTCACCCTACTCTCGGGCT -3'	202
RPLP0	Forward 5'- GCAGCATCTACAACCCTGAA -3'<br>Reverse 5'- GCAGATGGATCAGCCAAGAA -3'	235
SNAP25	Forward 5'- GTCACATGGCCCTGGATATGG -3'<br>Reverse 5'- CACTTAACCACTTCCCAGCAT -3'	143
TGF-β2	Forward 5'- GTACTACGCCAAGGAGGTTTAC -3'<br>Reverse 5'- TGTGGAGGTGCCATCAATAC -3'	568
TSLP	Forward 5'- AAGGCAACAGCATGGGTGAA -3'<br>Reverse 5'- TACGTGGACACCCAATTCCA -3'	160
TSP1	Forward 5'- AAAGGATAATTGCCCCAACC -3'<br>Reverse 5'-CGGTCTCCCACATCATCTCT -3'	177
VCAM-1[6]	Forward 5'- CGTCTCCATTGACTTGCAGCACC -3'<br>Reverse 5'- GTGATCGGCTTCCCAGCCTC -3'	276

*References for primers sequences sourced from the literature are provided below. For primer pairs designed in-house, products were confirmed by sequencing.

ANGTPL7= angiopoietin-like 7; BIRC3= baculoviral IAP repeat containing 3; FGF2= fibroblast growth factor 2; ICAM1= intercellular adhesion molecule 1; Linc= long intergenic non-protein coding RNA; IL = interleukin; LRP8= low-density lipoprotein receptor-related protein 8; MALAT1= metastasis associated lung adenocarcinoma transcript 1; MIR155HG= MIR155 host gene; NEAT1= nuclear paraspeckle assembly transcript 1; NFκB1= nuclear factor kappa B subunit 1; PPIA= peptidylprolyl isomerase A; PTGS2= prostaglandin-endoperoxide synthase 2; RPLP0= ribosomal protein lateral stalk subunit P0; SNAP25= synaptosomal-associated protein 25; TGF-β2 = transforming growth factor β2; TSLP= thymic stromal lymphopoietin; TSP1 = thrombospondin 1; VCAM-1: vascular cell adhesion molecule 1.

References

1. Nio-Kobayashi J, Kudo M, Sakuragi N, Kimura S, Iwanaga T, Duncan WC. Regulated C-C motif ligand 2 (CCL2) in luteal cells contributes to macrophage infiltration into the human corpus luteum during luteolysis. Mol Hum Reprod. 2015;21:645-654.

2. Blengio F, Raggi F, Pierobon D, et al. The hypoxic environment reprograms the cytokine/chemokine expression profile of human mature dendritic cells. Immunobiology. 2013;218:76-89.

3. Jang CH, Choi JH, Byun MS, Jue DM. Chloroquine inhibits production of TNF-alpha, IL-1beta and IL-6 from lipopolysaccharide-stimulated human monocytes/macrophages by different modes. Rheumatology. 2006;45:703-710.

4. Lu Y, Fukuda K, Nakamura Y, Kimura K, Kumagai N, Nishida T. Inhibitory effect of triptolide on chemokine expression induced by pro-inflammatory cytokines in human corneal fibroblasts. Invest Ophthalmol Vis Sci. 2005;46:2346-2352.

5. Rao NA, McCalman MT, Moulos P, et al. Coactivation of GR and NFKB alters the repertoire of their binding sites and target genes. Genome Res. 2011;21:1404-1416.

6. Smith JR, Choi D, Chipps TJ, et al. Unique gene expression profiles of donor-matched human retinal and choroidal vascular endothelial cells. Invest Ophthalmol Vis Sci. 2007;48:2676-2684.

Supplementary Table S2. Primer kits for microRNA (MystiCq MicroRNA Quantification System, Sigma-Aldrich Merck, St. Louis, MO).

MicroRNA	Catalogue number	Mature sequence
miR-10b-5p	MIRAP00023	UACCCUGUAGAACCGAAUUUGUG
miR-449c-3p	MIRAP00404	UUGCUAGUUGCACUCCUCUCUGU
miR-449b-3p	MIRAP00402	CAGCCACAACUACCCUGCCACU
miR-3130-5p	MIRAP00863	UACCCAGUCUCCGGUGCAGCC
miR-670-5p	MIRAP00662	GUCCCUGAGUGUAUGUGGUG
miR-212-3p	MIRAP00264	UAACAGUCUCCAGUCACGGCC
miR-146b-5p	MIRAP00185	UGAGAACUGAAUUCCAUAGGCU
miR-191-5p	MIRAP00226	CAACGGAAUCCCAAAAGCAGCUG
SNORD44	MIRCP00005	CCUGGAUGAUGAUAAGCAAAUGCUGACUGAACAUGAAGGUCUUAAUUAGCUCUAACUGACU

Supplementary Table S3. Alignment statistics for RNA-Sequencing on total and small RNA extracted from *T. gondii*-infected or uninfected human retinal pigment epithelial cells.

	**Status**	**Donor 1**		**Donor 2**		**Donor 3**		**ARPE19-1**	
		**Ctrl**	***T. gondii***	**Ctrl**	***T. gondii***	**Ctrl**	***T. gondii***	**Ctrl**	***T. gondii***
Total RNA	%mapped	35.71	17.62	73.07	35.73	52.64	46.94	79.95	34.76
	% Assigned to genes	71.46	58.36	71.81	66.32	71.81	72.95	69.96	65.54
Small RNA	%mapped	0.26	0.11	0.16	0.17	0.24	0.15	0.26	0.15
	% Assigned to genes	0.74	0.57	0.61	0.58	0.66	0.57	0.64	0.63

Supplementary Table S10. Relative normalized expression by RT-qPCR of selected protein-coding and long non-coding transcripts expressed by *T. gondii*-infected or uninfected human retinal pigment epithelial cells (presented with corresponding fold-change by RNA-sequencing).

**Molecule**	**RNA-seq**	**RT-qPCR**					
		**Donor 4**		**Donor 5**		**Donor 6**	
		**FC**	**p-value**	**FC**	**p-value**	**FC**	**p-value**
**ANGPTL7**	-9.2	-14.9	$1.3x10^{-2}$	-29.3	$1.3x10^{-5}$	-4.2	$1.1x10^{-3}$
**BIRC3**	62.2	312.7	$6.6x10^{-3}$	56	$4.1x10^{-5}$	295.2	$9.2x10^{-3}$
**CCL2**	4.21	10	$2x10^{-6}$	18.5	$1.3x10^{-3}$	8.9	$1.4x10^{-4}$
**CXCL3**	7.6	8.4	$1.7x10^{-2}$	45.7	$2x10^{-3}$	19.1	$5.5x10^{-2}$
**CXCL8**	5.6	7.7	$4.1x10^{-3}$	8.6	$2.5x10^{-5}$	[1.4]	NS
**FGF2**	-2.8	[1.0]	NS	-3.1	$2.6x10^{-2}$	-1.5	$7.1x10^{-2}$
**ICAM-1**	5.2	4.6	$4.2x10^{-4}$	3.1	$1.9x10^{-3}$	25.5	$3.3x10^{-4}$
**IL1RN**	149.5	882.1	$1.3x10^{-5}$	243.2	$5.2x10^{-4}$	5282.4	$2.8x10^{-2}$
**IL-6**	6.3	60.3	$4.5x10^{-3}$	82.4	$7.3x10^{-4}$	77.8	$1.6x10^{-3}$
**IL-11**	5.8	12.9	$4.7x10^{-4}$	15.9	$4.5x10^{-3}$	7	$1.2x10^{-4}$
**IL-12A**	-5.2	-4.1	$8.9x10^{-3}$	-10.4	$1.4x10^{-3}$	-7	$8.8x10^{-4}$
**IL17RB**	49.9	45.9	$1.3x10^{-4}$	165.9	$7.8x10^{-4}$	267.4	$1.3x10^{-2}$
**LINC00968**	-8.8	-5.7	$3.4x10^{-2}$	-5	$7.8x10^{-4}$	-5.3	$7.3x10^{-3}$
**LINC01105**	-5.3	-8	$3.5x10^{-3}$	-8.9	$7x10^{-6}$	-8.9	$2.3x10^{-2}$
**LRP8**	6.9	4.9	$6.6x10^{-3}$	11.2	$3.6x10^{-3}$	11.3	$1.9x10^{-3}$
**MALAT1**	-2.3	-2.5	$2x10^{-2}$	-2.3	$3.8x10^{-2}$	-2.6	$3.5x10^{-2}$
**MIR155HG**	14.2	14.2	$8x10^{-6}$	5.3	$5.3x10^{-4}$	12.6	$6.2x10^{-5}$
**NEAT1**	-3.1	-3.4	$9.4x10^{-3}$	-3	$4.2x10^{-2}$	[-1.2]	NS
**NFκB1**	3.7	4.1	$4.1x10^{-2}$	2.4	$7.1x10^{-2}$	5.24971	$7.4x10^{-3}$
**PTGS2**	37.4	13.7	$5.1x10^{-3}$	20.8	$1.4x10^{-3}$	6.9	$3.9x10^{-3}$

**RP11-572C15.6**	-7.3	-4.3	$4.6x10^{-2}$	-6.1	$6x10^{-6}$	[-1.1]	NS
**SNAP25**	49.1	114.1	$9.3x10^{-3}$	34.3	$2.3x10^{-4}$	40.5	$2.4x10^{-4}$
**TGF-β2**	-3.1	-2.7	$6.5x10^{-2}$	-3.8	$1.3x10^{-5}$	-2.2	$5.0x10^{-2}$
**TSLP**	17.3	69.9	$5.6x10^{-2}$	45.7	$1.7x10^{-4}$	47.5	$9x10^{-5}$
**TSP1**	-3.7	-2.6	$4.4x10^{-2}$	-2.5	$2.4x10^{-4}$	-2.5	$3.1x10^{-2}$
**VCAM-1**	15.7	14.3	$6.8x10^{-4}$	[-1.7]	$1.3x10^{-2}$	[1.2]	NS

Abbreviations: FC= fold-change; NS= not significant; ANGTPL7= angiopoietin-like 7; BIRC3= baculoviral IAP repeat containing 3; FGF2= fibroblast growth factor 2; ICAM1= intercellular adhesion molecule 1; Linc= long intergenic non-protein coding RNA; IL = interleukin; LRP8= low-density lipoprotein receptor-related protein 8; MALAT1= metastasis associated lung adenocarcinoma transcript 1; MIR155HG= MIR155 host gene; NEAT1= nuclear paraspeckle assembly transcript 1; NFκB1= nuclear factor kappa B subunit 1; PTGS2= prostaglandin-endoperoxide synthase 2; SNAP25= synaptosomal-associated protein 25; TGF-β2 = transforming growth factor β2; TSLP= thymic stromal lymphopoietin; TSP1 = thrombospondin 1; VCAM-1: vascular cell adhesion molecule 1.

Supplementary Table S11. Relative normalized expression by RT-qPCR of selected microRNA expressed by *T. gondii*-infected or uninfected human retinal pigment epithelial cells (presented with corresponding fold-change by RNA-sequencing).

Molecule	Strand	RNA-seq	RT-qPCR					
			Donor 4		Donor 5		Donor 6	
			FC	p-value	FC	p-value	FC	p-value
**miR-670**	+	169.9	[2.0]	NS	[1.5]	NS	3.7	6.5×10^{-2}
**miR-146b**	+	13.2	4.1	8.3×10^{-3}	3.7	6.7×10^{-4}	[1.2]	NS
**miR-10b**	+	12.2	[-1.0]	NS	[-1.3]	NS	[-1.3]	NS
**miR-543**	+	19.4						
**miR-449c**	-	7.5	-1.7	3.8×10^{-2}	[1.1]	NS	[2.1]	NS
**miR-449b**	-	13.4	[-1.6]	NS	[-1.4]	NS	[1.0]	NS
**miR-212**	-	6.6	8.2	7.2×10^{-4}	1.8	2.2×10^{-2}	6.7	3.9×10^{-4}
**miR-3130-1**	+	-45.0	[-1.4]	NS	[-1.3]	NS	2.1	6.5×10^{-4}

Abbreviations: FC= fold-change; NS= not significant
